# Structure-Activity Relationship of Benzophenanthridine Alkaloids from *Zanthoxylum rhoifolium* Having Antimicrobial Activity

**DOI:** 10.1371/journal.pone.0097000

**Published:** 2014-05-13

**Authors:** Luciana de C. Tavares, Graciane Zanon, Andréia D. Weber, Alexandre T. Neto, Clarice P. Mostardeiro, Ivana B. M. Da Cruz, Raul M. Oliveira, Vinicius Ilha, Ionara I. Dalcol, Ademir F. Morel

**Affiliations:** 1 Center for Research in Natural Products (NPPN), Federal University of Santa Maria, Santa Maria, RS, Brazil; 2 Graduate Program in Pharmacology, Health Sciences Center, Federal University of Santa Maria, Santa Maria, RS, Brazil; 3 Graduate Program in Toxicological Biochemistry, Federal University of Santa Maria, Santa Maria, RS, Brazil; Centro Nacional de Biotecnologia – CSIC, Spain

## Abstract

*Zanthoxylum rhoifolium* (Rutaceae) is a plant alkaloid that grows in South America and has been used in Brazilian traditional medicine for the treatment of different health problems. The present study was designed to evaluate the antimicrobial activity of the steam bark crude methanol extract, fractions, and pure alkaloids of *Z. rhoifolium*. Its stem bark extracts exhibited a broad spectrum of antimicrobial activity, ranging from 12.5 to 100 µg/mL using bioautography method, and from 125 to 500 µg/mL in the microdilution bioassay. From the dichloromethane basic fraction, three furoquinoline alkaloids (**1–3**), and nine benzophenanthridine alkaloids (**4–12**) were isolated and the antimicrobial activity of the benzophenanthridine alkaloids is discussed in terms of structure-activity relationships. The alkaloid with the widest spectrum of activity was chelerythrine (**10**), followed by avicine (**12**) and dihydrochelerythrine (**4**). The minimal inhibitory concentrations of chelerythrine, of 1.50 µg/mL for all bacteria tested, and between 3.12 and 6.25 µg/mL for the yeast tested, show this compound to be a more powerful antimicrobial agent when compared with the other active alkaloids isolated from *Z. rhoifolium*. To verify the potential importance of the methylenedioxy group (ring A) of these alkaloids, chelerythrine was selected to represent the remainder of the benzophenanthridine alkaloids isolated in this work and was subjected to a demethylation reaction giving derivative **14**. Compared to chelerythrine, the derivative (**14**) was less active against the tested bacteria and fungi. Kinetic measurements of the bacteriolytic activities of chelerythrine against the bacteria *Bacillus subtilis* (Gram-positive) and *Escherichia coli* (Gram-negative) were determined by optical density based on real time assay, suggesting that its mechanism of action is not bacteriolytic. The present study did not detect hemolytic effects of chelerythrine on erythrocytes and found a protective effect considering the decrease in TBARS and AOPP (advanced oxidized protein products) levels when compared to the control group.

## Introduction

Many studies have underlined the importance and use of plants as a source of antimicrobial agents [1]. In this context, plants of the genus Zanthoxylum (Rutaceae), which encompasses about 250 species distributed throughout the world, have been of great importance. Among these, *Zanthoxylum rhoifolium* (syn. *Fagara rhoifoliu*m), native to South America (Brazil, Uruguay, Paraguay and Argentina) has been used in Brazilian traditional medicine for the treatment of many different health problems [2]–[4]. In French Guiana, *Z. rhoifolium* has been used as an antimalarial treatment [5]. These plants are known to be a rich source of natural products, mainly alkaloids and lignans. Some of these alkaloids, including the benzophenanthridines and furoquinolines, display a variety of biological activities, such as antitumor [6]–[11], antimicrobial [12], anti-inflammatory [13]–[15] and antiplasmodial [16]–[18] activity.

In previous studies of our research team on the crude extract of *Z. rhoifolium*, the isolation and structural elucidation of a number of alkaloids have been reported, including zanthoxyline, nitidine, oxynitidine and skimmianine [19]. We have also reported the antibacterial activity of the crude MeOH extract, aqueous extract, acid and basic fractions of *Z. rhoifolium* by disc diffusion method [20] and of some isolated alkaloids by the TLC bioassay (bioautography method) [21]. More recently, our research team reported the presence of several benzophenanthridine alkaloids with interesting antitumoral activities from the same source [22]. Because many diseases that are widespread among underprivileged and indigenous populations, such as fever, stomach upset and respiratory diseases, among others, can be caused by fungi and bacteria, this study investigated the antimicrobial activities of the crude methanol extract, fractions obtained from this extract and purified compounds from the stem bark of *Z. rhoifolium* collected in Rio Grande do Sul, Brazil, were initially screened by bioautography method [23], [24] to select extracts, fractions and active alkaloids. The selected extracts, fractions and alkaloids were subsequently analyzed using the broth microdilution method [25], [26] for determining the minimum inhibitory concentration (MIC) and the minimum bactericidal/fungicidal concentration (MBC/MFC). Chelerythrine, representing the other benzophenanthridine alkaloids isolated in this work, presented potential anti-tumor, anti-microbial and anti-inflammatory properties [27]. Chelerythrine is also a well-known protein kinase C inhibitor molecule responsible for the maintenance of erythrocyte deformability [28]. Some studies, such as that of Kim et al. [29], have suggested that chelerythrine stimulates the production of reactive oxygen species.

**Figure 1:**
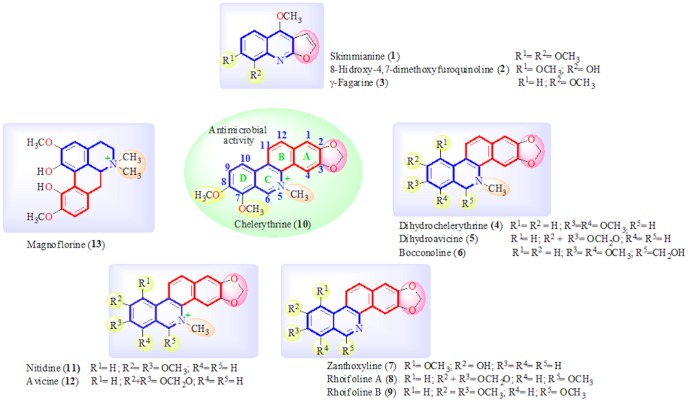
Alkaloids isolated from stem bark of *Zanthoxylum rhoifolium*.

The acid and basic fractions of the plant, obtained after acid-basic extraction of part of the methanol crude extract, exhibited significant inhibitory activity against some of the microorganisms tested. A bioassay-guided study of the dichloromethane (DCM) basic fraction led to the isolation and identification of three furoquinoline alkaloids, skimmianine (**1**), 8-hydroxy-4,7-dimetoxy-furoquinoline (**2**), and γ-fagarine (**3**), and nine benzophenanthridine alkaloids, dihydrochelerythrine (**4**), dihydroavicine (**5**), bocconoline (**6**), zanthoxyline (**7**), rhoifoline A (**8**), rhoifoline B (**9**), chelerythrine (**10**), nitidine (**11**) and avicine (**12**) and one aporphine-type of isoquinoline alkaloid, magnoflorine (**13**), (Figure 1). As well as, test here if the erythrocytes exposition to chelerythrine (**10**) could to triggered hemolysis and oxidative stress.

## Materials and Methods

### General experimental procedures

Melting points were determined in a MQAPF-301 melting point apparatus and are uncorrected. NMR spectra were acquired on a Brüker DPX-400 operating at 400 and 100 MHz, for ^1^H and ^13^C, respectively. Chemical shifts are given in δ (ppm) using TMS as internal standard. HR-mass spectra were recorded on a Brüker BioApex 70ev mass spectrometer. TLC was performed on pre-coated silica gel 60 F _254_ plates (Merck) and detection was achieved by UV light (254 nm), by spraying with 10% H_2_SO_4_, followed by heating. Chloramphenicol, ampicillin, azithromycin and nystatin were purchased from the Sigma-Aldrich Chemical Co, were used as control antibiotics.

### Plant material

*Z. rhoifolium* was collected in Santana do Livramento, (latitude 30°53′27" South and longitude 55°31′58" west), Rio Grande do Sul, Brazil, in November 2008, and authenticated by Dr. Gilberto D. Zanetti, Department of Pharmacy, Universidade Federal de Santa Maria, RS, Brazil, where a specimen sample (HDFI 134) is retained *Z. rhoifolium* was collected at a private property of Mr. Danilo Farias Morel. The species under study was confirmed to not be on the list of endangered species. Furthermore, our research group is authorized to collect species of the Rutaceae family (Access Authorization and Dispatch of Component Genetic Heritage Process-CNPq 010777/2013-0).

### Extraction and isolation procedures

The air-dried stem bark of *Z. rhoifolium* was pulverized in a wiley mill to powder form (1 Kg) and extracted four times with MeOH at room temperature. At this stage, the formation of an insoluble precipitate was observed, which was recovered by filtration (IP, 2.5 g) and saved for later analysis. The MeOH extract was filtered and concentrated in vacuum to obtain a crude extract (ME, 150 g). Part of the residual syrup (50 g) was dissolved in water and extracted with n-hexane (HF, 6.5 g), CH_2_Cl_2_ (DCM, 2.5 g), EtOAc (AF, 2.5 g) and n-butanol (BuF, 7.5 g), successively. The DCM fraction (2.0 g) containing divers Dragendorff's-positive spots in TLC, was chromatographed over silica gel (150 g) using CH_2_Cl_2_ with an increasing amount of MeOH, to prepare fractions 1–30 (200 mL/fraction). Fraction 3 (CH_2_Cl_2_, 100%), consisting of one alkaloid, was concentrated in vacuum to give skimmianine (**1**, 10.5 mg). Fraction 6 (CH_2_Cl_2_-MeOH, 99∶1), consisting of two alkaloids, was concentrated in vacuum (30.0 mg) and submitted to preparative TLC (CH_2_Cl_2_: MeOH, 99∶1) to yield γ-fagarine (**3**, 8.0 mg) and 8-hydroxy-4, 7-dimetoxy-furoquinoline (**2**, 9.0 mg). Fraction 9 (CH_2_Cl_2_: MeOH 99∶1), consisting of one alkaloid, was concentrated in vacuum to give zanthoxyline (**7**, 6.0 mg). Fractions 12–13 (CH_2_Cl_2_: MeOH, 98∶2) were combined (104.0 mg) and resubmitted to a silica gel column (10 g) eluted with CH_2_Cl_2_ containing increasing amounts of MeOH (up to 3%) to give dihydrochelerythrine (**4**, 21.0 mg) and dihydroavicine (**5**, 32.0 mg). Fractions 25–26 (CH_2_Cl_2_: MeOH, 80∶20) were combined (287.0 mg) and crystallized from MeOH-diethyl ether to give chelerythrine (**10**, 150.0 mg). The insoluble precipitate (IP, 0.5 g), was dissolved in MeOH: H_2_O and loaded onto a 7.5×12 cm C-18 column. The column was eluted with MeOH: H_2_O (60∶40 to 100∶0). Fraction 3 and 4, consisting of one alkaloid, were combined (50.0 mg) and submitted to preparative TLC (EtOAc: MeOH, 2∶3) to give magnoflorine (**13**, 10.0 mg), not analyzed in this work.

Part of the MeOH extract (70.0 g) was dissolved in H_2_O (100 mL) and acidified with 2M HCl to pH 2–3. After exhaustive extraction with n-hexane and Et_2_O, the acidic solution was made basic with NH_4_OH to pH 8–9 and extracted with DCM (5×100 mL) to yield the basic extract (4.5 g). The DCM basic fraction (4.0 g) was chromatographed on a silica gel column (230–400 mesh, 320 g) and eluted with CH_2_Cl_2_: MeOH mixture to prepare fractions 1–90. Fractions 3–8, obtained from CH_2_Cl_2_: MeOH 0.5% and consisting of three alkaloids, were combined (90.0 mg) and submitted to preparative TLC (CH_2_Cl_2_: MeOH, 99∶1, two elutions) to yield skimmianine (**1**, 20.2 mg), γ-fagarine (**3**, 11.0 mg) and zanthoxyline (**7**, 10.0 mg). Fraction 9 (CH_2_Cl_2_: MeOH 0.5%) was evaporated to give 8-hydroxy-4, 7-dimetoxy-furoquinoline (**2**, 12.0 mg). Fractions 11–20 obtained from CH_2_Cl_2_: MeOH 1%, were combined (350.0 mg) and resubmitted to a silica gel column (30 g) eluted with CH_2_Cl_2_ containing increasing amounts of MeOH (up to 3%) to give dihydrochelerythrine (**4**, 30.0 mg), dihydroavicine (**5**, 12.0 mg), rhoifoline A (**8**, 21.0 mg) and rhoifoline B (**9**, 19.5 mg). Fraction 21, obtained from CH_2_Cl_2_: MeOH, 98∶2, was evaporated to give bocconoline (**6**, 4.5 mg). Fractions 35–38 obtained from CH_2_Cl_2_: MeOH, 80∶20, and consisting of one alkaloid (TLC), were combined and concentrated in vacuum to give chelerythrine (**10**) after crystallization from Et_2_O-MeOH (120.0 mg). Fractions 45–48 obtained from CH_2_Cl_2_: MeOH, 70∶30, which contained two Dragendorff's-positive spots in TLC, were combined and concentrated in vacuum (129.0 mg) and submitted to a silica gel column (230–400 mesh) using EtOAc: MeOH (100∶0 →95∶5 →90∶10→80∶20) to give avicine (**11**, 25.0 mg) and nitidine (**12**, 22.0 mg).

Alkaloids **1**, **2**, **3**, **11, 12** and **13** were identified by comparison of their spectral data (EIMS, ^1^H- and ^13^C-NMR) with reported values in the literature [21], [30]–[32]. Alkaloids **4–10** were identified by direct comparison of TLC with authentic samples of zanthoxyline, dihydrochelerythrine, rhoifoline A, rhoifoline B, bocconoline, chelerythrine, respectively, and by comparison of their spectral data (^1^H- and ^13^C-NMR) with reported values in the literature [21].

### Preparation of 2,3-dihydroxy chelerythrine (14)

Chelerythrine (**10**, 0.20 mg) in dry dichloromethane (3 mL) was stirred at −20°C under argon atmosphere for 30 min. Boron tribromide (1 M, in dichloromethane, 5 mL) was added to the mixture which was then stirred during 18 h. The reaction was quenched by addition of methanol (10 mL). The solvent was removed in vacuum and the residue was purified by reverse phase liquid chromatography from methanol, to yield the 2,3-dihydroxy chelerythrine derivative (**14**) in 33% yield, as a yellowish oil: ^1^H-NMR (400MHz, DMSO-*d*_6_) *δ*: 9.86 (s, 1H), 8.62 (d, *J* = 9.6 Hz, 1H), 8.50 (d, *J* = 9.6 Hz, 1H), 8.15 (d, *J* = 9.6 Hz, 1H), 7.80 (d, *J* = 9.6 Hz, 1H), 7.30 (s, 1H), 7.07 (s, 1H), 4.65 (s, 3H), 4.04 (s, 3H), 3.47 (s, 3H). HREIMS *m/z* = 336.1219 (calcd for C_20_H_18_NO_4_, 336.1230).

### Antimicrobial test methods

The antimicrobial activity of *Z. rhoifolium* (extracts, isolated alkaloids and chelerythrine derivative) was tested against seven Gram-positive bacteria: *Bacillus subtilis* ATCC 6633, *Bacillus cereus* ATCC 33019, *Staphylococcus aureus* ATCC 25923. *Staphylococcus epidermidis* ATCC 12228, *Streptococcus pyogenes* ATCC 19615, *Enterobacter aerogenes* ATCC 13048, *Enterococcus spp*. ATCC 6589; eight *Gram-negative bacteria: Escherichia coli* ATCC 25922, *Klebsiella pneumonia* ATCC 13883, *Pseudomonas aeruginosa* ATCC 27853, *Enterobacter cloacae* ATCC 1304, *Shigella sonnei* ATCC 25931, *Salmonella typhimurium* ATCC 14028, *Burkholderia cepacia* ATCC 17759, *Morganella morganii* ATCC 25829; and seven yeasts: *Candida albicans* ATCC 10231, *Candida tropicalis* ATCC 18803 *Candida krusei* ATCC 6258, *Candida parapslosis* ATCC 22018, *Sacharomyces cerevisae* ATCC 2601, *Cryptococcus neoformans* ATCC 28952, *Cryptococcus gatti* ATCC 2601.

#### Bioautography method

For the antimicrobial assay, 100.0, 50.0, 25.0, 12.5, 6.25, 3.12, 1.56, 0.75 and 0.15 µg of extracts and isolated alkaloids were applied to pre-coated TLC plates [22], [23]. TLC plates were developed (only for isolated alkaloids) with CH_2_Cl_2_: MeOH 95∶5, and dried for complete removal of solvents. Mueller-Hinton agar medium (MHA-Merck) was inoculated with suspension microorganisms in saline solution (10^5^ CFU/mL) and distributed over TLC plates. The plates were incubated for 24 hours at 37°C and 72 hours at 25°C for bacteria and yeasts, respectively. The standard antibiotics chloramphenicol and nystatin were used as control and reference. Results were obtained by staining with an aqueous solution of 2, 3, 5-triphenyl-tetrazolium chloride (TTC, 1 mg/mL). The appearance of inhibition zones was used to demonstrate the least amount of a sample that was capable of inhibiting the growth of the microorganisms. All samples were tested in triplicate.

#### Broth Microdilution Method

The minimal inhibitory concentration (MIC) was determined on 96 well culture plates by a microdilution method using a microorganism suspension at a density of 10^5^ CFU.mL^−1^ with Casein Soy Broth incubated for 24 hours at 37°C for bacteria, and Sabouraud Broth incubated for 72 hours at 25°C for yeasts. The cultures that did not present growth were used to inoculate plates of solid medium (Muller Hinton Agar and Sabouraud Agar) in order to determine the minimum bactericidal/fungicidal concentration (MBC/MFC). Proper blanks were assayed simultaneously and samples were tested in triplicate. Technical data have been described previously [25], [26].

### Bacteriolytic activity

#### Bacteria and culture conditions

Bacterial cells were cultivated in casein soy broth at 37°C. The overnight bacterial culture cells were harvested by centrifugation (10 min, 3000 rpm), resuspended in fresh casein soy broth, and the number adjusted to approximately 5×10^7^ cells per mL (OD_600nm_µ0.2 with a SP-22 Spectrophotometer, Biospectro, Brazil). Bacterial culture in broth, solutions of ampicillin (positive control), and test samples were mixed in wells of a 96-well plate for optical density measurements.

#### Optical Density (OD) measurements

Bacterial culture (180 µL) was added in a 96-well microplate. The 96-well microplate was placed in a Spectra Max M2 microplate spectrophotometer (Molecular Devices Corp.) programmed to obtain measurements at 23°C. Optical densities of cell cultures were read using a 620 nm emission filter each 15 min with shaking (30 s) between each reading measurement. Cells were further cultivated to mid-logarithmic phase. Solutions of each test sample and antibiotic (20 µL) at different concentrations in fresh casein soy broth were added in a 96-well microplate. The 96-well microplate was placed in a Spectra Max M2 and OD of cell cultures containing test samples and antibiotic were read after 10 h of incubation. Technical data have been described previously [33], [34].

### Chelerythrine effect on erythrocyte hemolysis and oxidative stress

Cells used in this protocol were obtained from healthy volunteer donors from the Universidade Federal de Santa Maria (UFSM), in Rio Grande do Sul (RS), Brazil (age 32±5 years). This study is part of a project approved by the UFSM Ethical Board and all volunteers signed a term of consent. Blood samples were collected after 12 h overnight fasting by venipuncture, using gray and red top Vacutainers® (BD Diagnostics, Plymouth, UK) tubes with heparin. Specimens were routinely centrifuged within 1 h of collection for 15 min at 2500 g. The supernatant was discarded. The extent of hemolysis was determined as described in [35] with minor modifications. 1 mL of Na^+^/K^+^ phosphate buffered saline (PBS, pH 7.4) with 6 mM glucose was added to 1 mL of erythrocytes (1×10^9^). The chelerythrine was added concentrations of at 0.1 µM, 2 µM and 8 µM. The erythrocytes were incubated for 2 h and the erythrocyte count, hemolysis, lipoperoxidation and protein oxidation were evaluated and compared with the non-treated group. The concentration of hemoglobin was spectrophotometrically measured at 590 nm since in the supernatant indicates hemolysis. The absorbance value of 100% hemolysis was determined by adding 24 µL of erythrocytes to 4 mL PBS containing 0.01% Triton X-100.

Lipid peroxidation in human erythrocytes was quantified by measuring the formation of thiobarbituric acid-reactive substances (TBARS). Chelerythrine-exposed erythrocytes were washed twice with PBS and were mixed 1∶1 with 10% trichloroacetic acid. Samples were centrifuged for 10 min. Sodium chloride (150 µL, 150 mM), phosphoric acid (600 µL, 1%) and thiobarbituric acid (600 µL, 0.8%) were added to the supernatant. Samples were incubated at 100°C for 60 min. The amount of TBARS produced was measured at 532 nm and MDA was used to construct the standard curve. TBARS was used to measure lipoperoxidation in erythrocyte concentrations of 10^7^ cells [36]. The protein oxidation was evaluated by determination of advanced oxidized protein products (AOPP), measured in red blood cells using a Cobas Mira Plus clinical chemistry analyzer as described by Selmeci et al. [37].

The results obtained from the number of erythrocytes, hemolysis, lipoperoxidation and protein oxidation were expressed as % of non-treated control group. All experiments were performed in triplicate.

### Statistical analysis

Comparison among groups was performed by one-way analysis of variance followed by Dunnet *post hoc* test using GraphPad Prism software. Differences at p≤0.05 were considered significant.

## Results and Discussion

Over the years, our research team has studied the species *Z. rhoifolium*, one of the most popular species of plants used in southern Brazil for a series of illnesses. Although not commonly recognized as an antimicrobial species, its popular use for ailments such as fever and stomach problems, among others, may be associated with the treatment of microorganisms such as fungi and bacteria.

**Figure 2:**
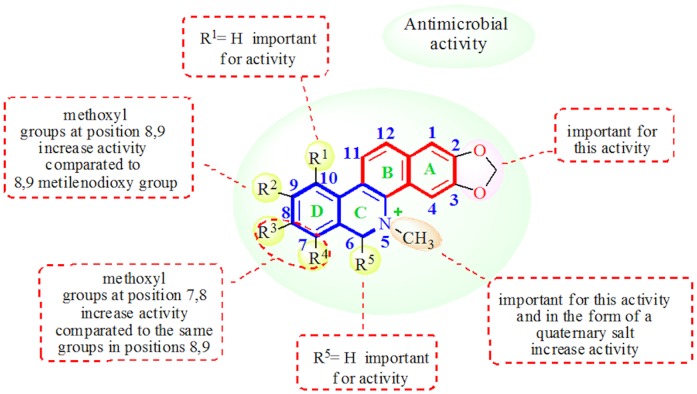
Figure illustrating the structure-activity relationship for the antimicrobial activity of benzophenanthridine alkaloids.

The present study reports the antimicrobial activities of extracts and compounds isolated from *Z. rhoifolium*, evaluated initially by means of bioautography bioassay [23], [24] to obtain the minimum amount required for inhibition of microbial growth on TLC plates in µg (Table 1). This test is valuable for bioassay-guided fractionations and for activity comparisons for compounds of similar diffusion coefficients in the TLC plate material. The results show that the DCM basic fraction and the alkaloids, dihydrochelerythrine (**4**), dihydroavicine (**5**), bocconoline (**6**) and chelerythrine (**10**) presented activity against all strains of bacteria tested. Dihydrochelerythrine (**4**) and dihydroavicine (**5**) were active against *Candida albicans*, while chelerythrine (**10**) was active against the three yeasts tested. Rhoifoline A (**8**) and rhoifoline B (**9**) were inactive against all strains tested. From the single isolated, the compound with the widest spectrum of activity was chelerythrine (**10**), followed by bocconoline (**6**), dihydrochelerythrine (**4**) and dihydroavicine (**5**). From the dihydroalkaloids **4–6**, dihydrochelerythrine (**4**), with two methoxyl groups at positions 7 and 8, was the most active. The weak activity of dihydroavicine (**5**), which features the methylenedioxy group at position 8–9, shows the importance of substitution at position 7 and 8 for this type of activity (Figure 2). From this result, the Minimal Inhibitory Concentration (MIC) was determined for the organisms which were sensitive to alkaloids **4**, **5** and **10** in the bioautography bioassay. Bocconoline (**6**) was not analyzed by this method, because of the small amount available. The benzophenanthridine alkaloids, zanthoxyline (**7**), nitidine (**11**) and avicine (**12**), and the furoquinoline alkaloids, skimmianine (**1**) and γ-fagarine (**3**), not analyzed by TLC bioassay, were then analyzed using the microdilution assay (Table 2).

**Table 1:** Minimum amount required (in µg) for inhibition of microbial growth on TLC plates for extracts and isolated alkaloids of *Z. rhoifolium*.

Microorganism										
Samples (µg)	Bacteria*									
	Gram (+)			Gram (−)				Yeasts^*^		
	*Se*	*Sa*	*Bs*	*Kp*	*Ec*	*Pa*	*Ss*	*Ca*	*Sc*	*Cn*
Dihydrochelerythrine (4)	3.12	3.12	3.12	3.12	1.56	1.56	1.56	25	12.5	NA
Dihydroavicine (5)	1.56	6.25	1.56	12.5	1.56	3.12	1.56	1.56	NA	NA
Bocconoline (6)	1.56	1.56	1.56	1.56	1.56	3.12	1.56	NA	NA	NA
Chelerythrine (10)	0.15	0.15	0.15	0.15	0.15	0.15	0.15	12.5	0.75	0.30
Methanol extrat	25	100	NT	25	12.5	NT	12.5	NT	NT	NT
CH_2_Cl_2_ fraction^a^	25	25	NT	25	25	NT	50	NT	NT	NT
Standard^b^	0.7	0.7	0.8	0.5	0.5	0.6	0.7	2.43	3.4	2.43

*(ATCC =  merican Type Culture Collection): Bacteria: Staphylococcus epidermidis-Se (ATCC 12228); Staphylococcus aureus-Sa (ATCC 6538); Bacillus subtilis-Bs (ATCC 6633); Klebsiella pneumoniae-Kp (ATCC 13883); Escherichia coli-Ec (ATCC 25922); Pseudomonas aeruginosa-Pa (ATCC 27853); *Shigella sonnei-Ss* (ATCC 25931); Yeasts: Candida albicans-Ca (ATCC 10231); Sacharomyces cerevisae-Sc (ATCC 2601); Cryptococcus neoformans-Cn (ATCC 28952). NT =  not tested; NA =  not active;

a basic;

b Chloramphenicol for bacteria and Nystatin for yeasts.

**Table 2:** Antimicrobial activity (MIC/MBC and MFC in µg/mL) for extracts and isolated alkaloids of *Z. rhoifolium*.

Microorganism																		
	Bacteria*																	
Samples (µg/mL)	Gram (+)								Gram (−)				Yeasts*					
	*Se*		*Sa*		*Sp*		*Bs*		*Kp*		*Ec*		*Ca*		*Sc*		*Cn*	
	MIC	MBC	MIC	MBC	MIC	MBC	MIC	MBC	MIC	MBC	MIC	MBC	MIC	MFC	MIC	MFC	MIC	MFC
Skimmianine (**1**)	100	>100	100	>100	100	>100	>100	>100	>100	>100	>100	>100	100	>100	100	>100	NT	NT
γ-Fagarine (**3**)	100	>100	50	>100	50	>100	100	>100	50	>100	100	>100	100	>100	100	>100	50	>100
Dihydrochelerythrine (4)	6.25	12.5	12.5	50	12.5	50	25	50	12.5	25	25	25	>100	>100	>100	>100	>100	>100
Dihydroavicine (**5**)	100	>100	100	>100	100	>100	100	>100	100	100	100	>100	>100	>100	>100	>100	>100	>100
Zanthoxyline (**7**)	50	50	50	50	50	>100	25	50	50	>100	50	>100	50	>100	100	>100	50	>100
Chelerythrine (**10**)	1.5	12.5	1.5	3.12	1.5	6.25	1.5	50	1.5	50	1.5	25	3.12	3.12	6.25	6.25	3.12	6.25
Nitidine (**11**)	>100	>100	25	50	>100	>100	>100	>100	50	50	>100	>100	12.5	25	6.25	25	25	>100
Avicine (**12**)	3.12	12.5	1.5	25	1.5	12.5	1.5	6.25	6.25	12.5	1.5	50	6.25	25	12.5	25	12.5	25
Methanol extrat	250	250	250	>500	250	>500	250	250	500	>500	500	>500	500	>500	>500	>500	>500	>500
CH_2_Cl_2_ fraction^a^	125	500	250	>500	250	>500	125	250	250	>500	125	>500	250	>500	250	>500	250	>500
Standard^b^	3.12	3.12	3.12	3.12	3.12	3.12	3.12	3.12	3.12	3.12	3.12	3.12	10.3	10.3	5.15	5.15	5.15	5.15

*(ATCC =  American Type Culture Collection): Bacteria: Staphylococcus epidermidis-Se (ATCC 12228); Staphylococcus aureus- Sa (ATCC 6538); Streptococcus pyogenes-Sp (ATCC 19615); Bacillus subtilis-Bs (ATCC 6633); Klebsiella pneumoniae-Kp (ATCC 13883); Escherichia coli-Ec (ATCC 25922); Yeasts: Candida albicans-Ca (ATCC 10231); Sacharomyces cerevisae-Sc (ATCC 2601); Cryptococcus neoformans-Cn (ATCC 28952). NT = not tested;

a basic;

b Chloramphenicol for bacteria and Nystatin for yeasts.

The minimal inhibitory concentrations of chelerythrine (**10**) for all bacteria and yeasts tested (MIC = 1.50 µg/mL, and between 3.12 and 12.5 µg/mL for bacteria and yeasts, respectively), demonstrate that this alkaloid has excellent antimicrobial activity compared to chloramphenicol (3.12 µg/mL for bacteria) and nystatin (between 5.15 to 10.3 µg/mL for yeasts). Avicine (**12**) was the second most active alkaloid, followed by dihydrochelerythrine (**4**) (Table 2). Unexpectedly, the alkaloid nitidine (**11**), with a structure very close to chelerythrine (**10**) and avicine (**12**), showed no significant growth inhibition against the bacteria tested. The main difference between the alkaloids nitidine (**11**) and avicine (**12**) is at carbons C-8 and C-9 of ring D. In nitidine (**11**), C-8 and C-9 are replaced with methoxyl groups, while in avicine (**12**), which showed great activity, C-8 and C-9 are substituted with a methylenedioxy group, showing the important contribution of this group at this position (Figure 2). This result is in agreement with the work of Miao et al. [38], who concluded that sanguinarine, with a methylenedioxy group at C-7 and C-8, exhibited a broader antibacterial spectrum than chelerythrine, with methoxyl groups at C-7 and C-8. On the other hand, nitidine (**11**) exhibited good antifungal activity against the yeast tested (MIC between 6.25–25 µg/mL). This result is in agreement with the results of Yang et al. [39], demonstrating that a 7, 8-methoxy group, in comparison to a 7, 8-methylenedioxy group, could enhance the antifungal activity to a certain degree. The same may be true for positions 8–9. Chelerythrine (**10**), the most active alkaloid of all, differs structurally from nitidine (**11**) in that it presents two methoxyl groups at C-7 and C-8. In addition, Table 2 shows that chelerythrine presented bactericidal activity against *Staphylococcus aureus* and *Streptococcus pyogenes* (MBC = 3.12 and 6.25 µg/mL, respectively), and fungicidal activity against all fungi tested (MFC between 3.12–6.25 µg/mL). The alkaloid avicine (**12**) also demonstrated bactericidal activity against *Bacillus subtilis* and *Klebsiella pneumoniae* (MBC = 6.25 and 12.5 µg/mL, respectively), and fungicidal activity against the fungi tested. Meanwhile, the alkaloid nitidine (**11**) presented fungicidal activity against *Candida albicans* and *Sacharomyces cerevisae* (MFC = 25 µg/mL). The alkaloid dihydrochelerythrine (**4**), although less active than the quaternary analogue base chelerythrine (**10**), presented bacteriostatic and bactericidal activity against all bacteria tested, but was inactive against the fungi tested (in this work bactericidal/fungicidal activity was considered for MBC/MFC at most 4×MIC values). The absence of antimicrobial activity for alkaloids **7–9** suggests that the nitrogen ring substituted with a methyl group, or in the form of a tertiary amine or a quaternary salt, is important for this kind of activity (Figure 2). Our results are in agreement with the results of Miao et al. [38] that prepared a series of alkoxyl and acetonyl derivatives at position 6 of sanguinarine and chelerythrine and examined their antibacterial activity. After analyzing the results, the researchers suggested that the double bond of C = N^+^ was determinant for the antibacterial activity of sanguinarine and chelerythrine.

**Figure 3:**
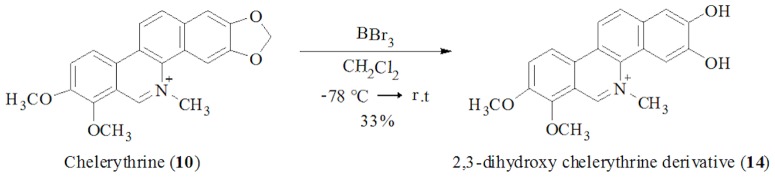
Reaction by obtention of 2,3-dihydroxy chelerythrine derivative (14) from Chelerythrine (10).

With the aim of observing the importance of the 2,3-methylenedioxy group (common in all isolated benzophenanthridine alkaloids) (Figure 2) in its antimicrobial activity, chelerythrine (**10**), representing the remaining benzophenanthridine alkaloids, was converted into its 2,3-dihydroxy derivative (**14**), by treatment with boron tribromide (Figure 3). Results of the antimicrobial activity of **14** (Table 3) show that derivative **14** is less active then the natural alkaloid chelerythrine (**10**) demonstrating the importance of the 2,3-methylenedioxy function for the antimicrobial activity of the isolated benzophenanthridine alkaloids. Among the furoquinoline alkaloids analyzed, only γ-fagarine (**3**) showed poor activity against *Staphylococcus aureus* (MIC = 50 µg/mL) and *Cryptococcus neoformans* (MIC = 50 µg/mL).

**Table 3:** Antimicrobial activity (MIC/MBC and MFC in µg/mL) for Chelerythrine (**10**) and 2,3-dihydroxy chelerythrine derivative (**14**).

	Compounds (µg/mL)				Standard (µg/mL)				
Microorganisms	Chelerythrine (10)		2,3-dihydroxy derivative (14)		Ampicillin		Azithromycin		
Bacteria* Gram (+)	**MIC**	**MBC**	**MIC**	**MBC**	**MIC**	**MBC**	**MIC**		**MBC**
*Sa*	1.5	3.12	50	100	0.77	50	0.77		50
*Bc*	12.5	25	50	50	50	50	1.55		1.55
*Bs*	1.5	6.25	25	>100	0.77	50	0.77		0.77
*Es*	3.12	>100	50	>100	25	50	1.55		6.25
*Ea*	50	>100	100	>100	50	50	12.5		12.5
Bacteria* Gram (-)									
*Ec*	1.5	25	>100	NT	3.12	25	1.55		3.12
*Pa*	2.5	25	25	>100	25	50	12.5		25
*E. cloacae*	50	>100	100	>100	50	50	0.77		1.55
*Ss*	25	100	100	>100	25	50	3.12		3.12
*St*	25	>100	50	>100	0.77	0.77	3.12		6.25
*B.cepacia*	3.12	100	50	>100	50	50	1.55		3.12
*Mm*	12.5	100	25	>100	0.77	6.25	3.12		6.25
Yeasts*					Nystatin				
	**MIC**	**MFC**	**MIC**	**MFC**	**MIC**			**MFC**	
*Ca*	3.12	3.12	12.5	50	0.77			3.12	
*Ct*	50	>100	12.5	50	1.52			3.12	
*Ck*	50	100	50	>100	0.77			6.25	
*Cp*	100	>100	100	>100	1.52			3.12	
*Sc*	6.25	6.25	12.5	50	1.52			3.12	
*Cn*	3.12	6.25	50	100	1.52			3.12	
*Cg*	6.25	25	100	>100	3.12			3.12	

*(ATCC =  American Type Culture Collection): Bacteria: Staphylococcus aureus- Sa (ATCC 6538); Bacillus cereus-Bc (ATCC 33019); Bacillus subtilis-Bs (ATCC 6633); *Enterococcus spp.-Es* (ATCC 6589); *Enterobacter aerogenes-Ea* (ATCC 13048); Escherichia coli-Ec (ATCC 25922); Pseudomonas aeruginosa-Pa (ATCC 27853); Enterobacter cloacae-E.cloacae (ATCC 1304); *Shigella sonnei-Ss (ATCC 25931); Salmonella typhimurium-St (ATCC 14028); Burkholderia cepacia-B.cepacia* (ATCC 17759); *Morganella morganii-Mm* (ATCC 25829); Yeasts: Candida albicans-Ca (ATCC 10231); Candida tropicalis-Ct (ATCC 18803); Candida krusei-Ck (ATCC 6258); Candida parapslosis-Cp (ATCC 22018); Sacharomyces cerevisae-Sc (ATCC 2601); Cryptococcus neoformans-Cn (ATCC 28952); Cryptococcus gatti-Cg (ATCC 2601). NT =  not tested; Ampicillin and Azithromycin for bacteria and Nystatin for yeasts.

**Figure 4:**
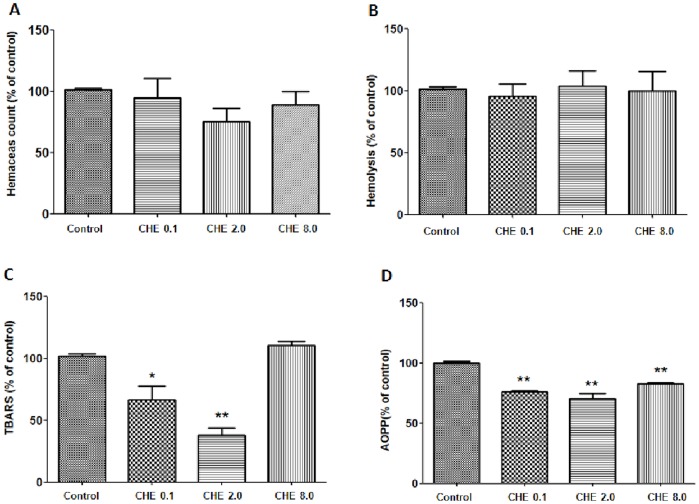
Chelerythrine (CHE) *in vitro* effect on human erythrocytes: (A) Erythrocytes count; (B) Hemolysis; (C) Lipoperoxidation evaluated by TBARS assay; (D) protein oxidation evaluated by Advanced Oxidative Protein Products (AOPP) assay. CHE treatments were compared to control group by one-way analysis of variance followed by Dunnet *post hoc* test. * = p<0.05; ** = p<0.01; *** = p<0.001.

Kinetic measurement of the bacteriolytic activities of chelerythrine (**10**) against the bacteria *Enterococcus* spp, *Bacillus subtilis*, *Bacillus cereus*, *Staphylococcus aureus* (Gram-positive) *Escherichia coli* and *Shigella sonnei* (Gram-negative) were determined by OD based on real time assay. Comparing the mode of action of chelerythrine (**10**) against the selected bacteria, we suggest that its mechanism of action is not bacteriolytic. Besides the bacteriostatic/bactericidal and fungistatic/fungicidal activities, previous studies published in the literature described chelerythrine (**10**) as presenting potential anti-tumor, anti-microbial and anti-inflammatory properties [27]. However, this molecule is also a well-known protein kinase C inhibitor that can act on peripheral and cytoskeleton proteins which are responsible for the maintenance of erythrocyte deformability [28]. In addition, some studies, such as that performed by Kim et al [29], have suggested that chelerythrine (**10**) stimulates the production of reactive oxygen species. Due to these data, an additional assay was performed to check whether chelerythrine (**10**) could trigger hemolysis and oxidative stress in human erythrocytes. As can be seen in Figure 4, different chelerythrine (**10**) concentrations did not affect the erythrocyte count or hemolysis when compared to the non-treated control group (Figure 4A, 4B). The evaluation of the effect of chelerythrine (**10**) on erythrocyte oxidative metabolism showed that at concentrations of 0.1 and 2.0 µM, a significant decrease in lipoperoxidation occurred in erythrocytes. At 8.0 µM, lipoperoxidation was similar to that observed in the control group (Figure 4C). Protein oxidation, evaluated by AOPP levels, also significantly decreased when compared to the control group (Figure 4D).

The present study did not detect a hemolytic effect of chelerythrine (**10**) on erythrocytes and found a protective effect considering the decrease in the TBARS and AOPP levels when compared to the control group. These results could be related to the concentrations of chelerythrine (**10**) to which erythrocytes were exposed. Chelerythrine (**10**) probably presented a dual effect on the oxidative metabolism since previous studies have described the cytotoxic effect of this molecule. Yamamoto et al. [40], for example, showed pyknosis, cytoplasmic vacuoles and cell shrinkage of cardiac myocytes exposed at ≥6 µM chelerythrine (**10**). On the other hand, Parlakpinar et al. [41] found a protective effect of chelerythrine (**10**) against nephrotoxicity caused by gentamicin (GEN) involving oxidative metabolism modulation. Gentamicin caused an increase in lipoperoxidation levels which decreased in the presence of chelerythrine (**10**). Therefore, our results suggest that chelerythrine (**10**) at concentrations lower than 8.0 µM did not cause toxicity and oxidative stress in human red blood cells.

In conclusion, based on analysis of the present results, it can be concluded that benzophenanthridine alkaloids are primarily responsible for the antimicrobial activity of *Z. rhoifolium*. In addition, the antimicrobial activity is related to the structure of the alkaloids. Quaternary alkaloids, such as chelerythrine (**10**) and avicine (**12**), are more active than the corresponding hydrogenated alkaloids, such as dihydrochelerythrine (**4**) and dihydroavicine (**5**). Furthermore, the type and position of substitution on ring D influences this type of activity. For example, comparing the activities of nitidine (**11**) and avicine (**12**), it can be observed that the methylenedioxy group in avicine (**12**) increases the antimicrobial activity when compared to that of nitidine (**11**) with two methoxyl groups in the same positions C-8 and C-9. Moreover, the position of the methoxyl groups is important for this activity, as can be seen in the comparison between the activities of nitidine (**11**) and chelerythrine (**10**). The two methoxyl groups at C-7 and C-8 (chelerythrine) increased the antimicrobial activity of the alkaloid when compared with that found for methoxyl groups at C-8 and C-9 (nitidine).

The influence of the methylenedioxy group in position 2-3 for this type of activity was evident for the chelerythrine derivative (**14**), resulting from methylenedioxy ring opening of chelerythrine (**10**), which showed a dramatic decrease in antimicrobial activity against some selected strains of bacteria and fungi. In summary, we can conclude that the species *Z. rhoifolium* is a very valuable plant, due to its secondary metabolites with potent pharmacological activities such as antitumor, antiplasmodial, anti-inflammatory and antimicrobial activity. The present study did not detect a hemolytic effect of chelerythrine (**10**) on erythrocytes and found a protective effect considering the decrease in the TBARS and AOPP levels when compared to the control group. Chelerythrine (**10**) probably presented a dual effect on the oxidative metabolism since previous studies have described the cytotoxic effect of this molecule. Therefore, our results indicate that chelerythrine (**10**) at concentrations lower than 8.0 µM did not cause toxicity and oxidative stress in human red blood cells.
